# Extracorporeal CO_2_ removal by hemodialysis: in vitro model and feasibility

**DOI:** 10.1186/s40635-017-0132-7

**Published:** 2017-04-07

**Authors:** Alexandra G. May, Ayan Sen, Matthew E. Cove, John A. Kellum, William J. Federspiel

**Affiliations:** 1 Department of Chemical Engineering, University of Pittsburgh, Pittsburgh, PA USA; 2 McGowan Institute for Regenerative Medicine, University of Pittsburgh, Pittsburgh, PA USA; 3 Department of Critical Care Medicine, University of Pittsburgh Medical Center, Pittsburgh, USA; 4 Department of Critical Care Medicine, Mayo Clinic Arizona, Phoenix, AZ USA; 5 Division of Respiratory and Critical Care Medicine, Department of Medicine, National University of Singapore, Level 10, 1E Kent Ridge Road, Singapore, 119228 Singapore; 6 Department of Bioengineering, University of Pittsburgh, Pittsburgh, PA USA

**Keywords:** Respiratory hemodialysis, Extracorporeal carbon dioxide removal, COPD, ARDS, ECCO_2_R

## Abstract

**Background:**

Critically ill patients with acute respiratory distress syndrome and acute exacerbations of chronic obstructive pulmonary disease often develop hypercapnia and require mechanical ventilation. Extracorporeal carbon dioxide removal can manage hypercarbia by removing carbon dioxide directly from the bloodstream. Respiratory hemodialysis uses traditional hemodialysis to remove CO_2_ from the blood, mainly as bicarbonate. In this study, Stewart’s approach to acid-base chemistry was used to create a dialysate that would maintain blood pH while removing CO_2_ as well as determine the blood and dialysate flow rates necessary to remove clinically relevant CO_2_ volumes.

**Methods:**

Bench studies were performed using a scaled down respiratory hemodialyzer in bovine or porcine blood. The scaling factor for the bench top experiments was 22.5. In vitro dialysate flow rates ranged from 2.2 to 24 mL/min (49.5–540 mL/min scaled up) and blood flow rates were set at 11 and 18.7 mL/min (248–421 mL/min scaled up). Blood inlet CO_2_ concentrations were set at 50 and 100 mmHg.

**Results:**

Results are reported as scaled up values. The CO_2_ removal rate was highest at intermittent hemodialysis blood and dialysate flow rates. At an inlet pCO_2_ of 50 mmHg, the CO_2_ removal rate increased from 62.6 ± 4.8 to 77.7 ± 3 mL/min when the blood flow rate increased from 248 to 421 mL/min. At an inlet pCO_2_ of 100 mmHg, the device was able to remove up to 117.8 ± 3.8 mL/min of CO_2_. None of the test conditions caused the blood pH to decrease, and increases were ≤0.08.

**Conclusions:**

When the bench top data is scaled up, the system removes a therapeutic amount of CO_2_ standard intermittent hemodialysis flow rates. The zero bicarbonate dialysate did not cause acidosis in the post-dialyzer blood. These results demonstrate that, with further development, respiratory hemodialysis can be a minimally invasive extracorporeal carbon dioxide removal treatment option.

## Background

Mechanically ventilating patients with acute respiratory distress syndrome (ARDS) can cause additional lung damage [1]. For ARDS patients, it is recommended to ventilate in accordance with lung protective ventilation (LPV) settings, tidal volumes limited to 6 mL/kg, and plateau pressures limited to 30 cmH_2_O [2]. Although an ARDS Network clinical trial demonstrated LPV reduced mortality by 8.8% [2], more recent studies have shown that these settings may still cause ventilator-induced lung injury (VILI) [3, 4]. To further reduce mortality, tidal volumes less than 6 mL/kg have been proposed [5]. Several studies have demonstrated the safety and feasibility of ultra-protective lung ventilation when used in conjunction with an extracorporeal CO_2_ removal (ECCO_2_R) device [5, 6].

In addition to ARDS, chronic obstructive pulmonary disease (COPD) patients frequently develop hypercapnia during acute exacerbations. Hypercapnia has long been recognized as a marker of poor prognosis in patients with COPD [1], with those requiring mechanical ventilation being particularly challenging to wean [7, 8]. A method to remove CO_2_ in these patients may obviate the need for mechanical ventilation altogether [9, 10]. When combined with the ARDS population, there is a significant unmet need for a simple, minimally invasive therapy to remove CO_2_.

Extracorporeal carbon dioxide removal is a technology that involves removal of blood from the patient, which is then pumped through an artificial lung (oxygenator membrane) where CO_2_ is removed and the decarboxylated blood is subsequently returned to the patient [1]. Conventional ECCO_2_R requires blood flow rates exceeding 1 L/min, as well as large surface areas for gas exchange in the artificial lung, both of which introduce important limitations. High flow rates require placement of large cannulas, which risk vessel injury and require considerable expertise to place [11]. Large surface areas in artificial lungs demand the use of anticoagulation, and bleeding complications have been high in previous ECCO_2_R trails [12]. In an attempt to mitigate some of these problems, low flow ECCO_2_R devices have been introduced, but they may take up to 24 h to control CO_2_ levels [10], and it has not yet been demonstrated in a clinical trial if these devices remove enough CO_2_ to support critically ill patients; however, several case reports and single site pilot studies have shown this [9, 10, 13–15].

A different approach involves using hemodialysis to remove CO_2_ in the form of bicarbonate. In respiratory hemodialysis, blood is passed through a dialyzer and bicarbonate is transferred from the blood to the dialysate based on a bicarbonate concentration difference between the two fluids. This is analogous to the movement of waste products in conventional hemodialysis. The similarities between respiratory and conventional hemodialysis provide additional benefits over membrane lung ECCO_2_R, including clinician familiarity, availability of dialysis equipment in intensive care units, and a reduced risk of air embolism. Bicarbonate removal, however, has proven challenging due to the development of metabolic acidosis, despite attempts to mitigate the acidosis by replacing the bicarbonate with bases, such as sodium hydroxide, and TRIS [1, 16, 17].

The attempt to “replace” bicarbonate, in these early experiments, reflects conventional acid-base teaching, where pH is dependent on, among other things, bicarbonate concentration. However, 30 years ago, Stewart proposed an alternative model of acid-base physiology based around the important observation that water has a dissociation constant and the principles of electrical neutrality [18]. This approach provides modeling advantages over the traditional acid-base approaches [19] by treating bicarbonate as a dependent anion. Stewart proposed that any compound, in solution, must satisfy both the principles of electrical neutrality and the respective dissociation constant, including water itself, where the dissociation constant (K’w) is equal to the product of hydrogen and hydroxyl ion concentrations ([H] × [OH]). Using simple algebra, Stewart showed plasma pH is dependent on the strong ion difference (SID) of the solution, the partial pressure of CO_2_ (pCO_2_), and the total concentration of weak acids (*A*_tot_). Similarly, the final bicarbonate concentration is dependent on the same variables, i.e., the concentration of bicarbonate does not predict pH, rather the SID, pCO_2_, and *A*_tot_, determine the final bicarbonate concentration. Therefore, a bicarbonate-free dialysis solution should remove bicarbonate from plasma, and allow it to return to a normal physiological pH as long as the strong ion difference and *A*_tot_ are maintained. In this manuscript, we explore whether ECCO_2_R, in the form of respiratory hemodialysis, is feasible without decreasing blood pH using a custom dialysate developed using Stewart’s model.

## Methods

### Construction of zero bicarbonate dialysate

The electrolyte composition of the dialysate was determined using a published acid-base model, pHorum [20]. This model uses the physicochemical approach to acid-base balance, which is based around the Stewart equation [21] shown below [22].

1 $$ \mathrm{S}\mathrm{I}\mathrm{D}=\left(\frac{K_{\mathrm{c}}*{\mathrm{pCO}}_2}{\left[{\mathrm{H}}^{+}\right]}+2\frac{K_{\mathrm{c}}*{K}_3*{\mathrm{pCO}}_2}{{\left[{\mathrm{H}}^{+}\right]}^2}+\frac{K_{\mathrm{w}}}{\left[{\mathrm{H}}^{+}\right]}+\frac{K_{\mathrm{a}}*\left[{A}_{\mathrm{tot}}\right]}{K_{\mathrm{a}}+\left[{\mathrm{H}}^{+}\right]}-\left[{\mathrm{H}}^{+}\right]\right) $$

*K*_c_ is the combined equilibrium and solubility constant for CO_2_ (2.45 × 10^−11^ mol^2^/(L^2^*mmHg)), *K*_3_ is the second dissociation constant of carbonic acid (5.76 × 10^−11^ mol/L), *K*_w_ is the autoionization constant of water applied to plasma (2.39 × 10^−14^ mol^2^/L^2^), *K*_a_ is the weak acid dissociation constant (1.77 × 10^−7^ mol/L), and *A*_tot_ is the total concentration of weak acids [23].

Using this model, one can predict the pH of blood under a range of circumstances. For the purposes of designing the dialysate, we determined the ideal electrolyte composition of blood that will maintain pH within the normal range after removal of bicarbonate and carbon dioxide. Physiologically, normal electrolyte concentrations were used as the starting inputs for the model. Table 1 below shows the analysis results, which represents the ideal electrolyte composition for blood exiting the dialysis filter following bicarbonate removal, assuming the bicarbonate concentration is <10 mmol/L and pCO_2_ is <20 mmHg. Since the sieving coefficient is close to 1.0 for most of these electrolytes, we used these concentrations to construct the dialysate. In order to maximize bicarbonate removal in our experiments, the bicarbonate concentration was kept at 0 mmol/L in the dialysate solution. The final pH of the modeled dialysis solution is 10, despite targeting a physiological SID, because the dialysate does not contain protein. The dialysate does not contain calcium to allow for the dialysate to be compatible with regional citrate anticoagulation. Furthermore, at a pH of 10 or more, there is a risk the calcium may precipitate out of solution.

**Table 1:** Ion concentrations in zero bicarbonate dialysate

	Normal plasma	Zero bicarbonate dialysate
pH^a^	7.35–7.45	10.0
Sodium (mmol/L) [18]	136–145	134
Potassium (mmol/L) [18]	3.5–5.0	3.5
Calcium^b^ (mmol/L) [18]	2.2–2.6	0
Magnesium (mmol/L) [18]	0.8–1.2	1.0
Chloride (mmol/L) [18]	98–106	116 (135.6 final concentration)^c^
Phosphate^d^ (mmol/L) [18]	2.0–4.5	0.5
Lactate (mmol/L)	<2.0	3.0
HCO_3_ (mmol/L)^a^	22–26	0

^a^For a pCO_2_ of 40 mmHg

^b^Total calcium

^c^Modelled chloride concentration was 116 mmol/L. HCl was added so that the dialysate could be analyzed on the blood gas machine resulting in a final concentration of 135.6 mmol/L

^d^PO_4_

### In vitro CO_2_ removal

**Fig. 1:**
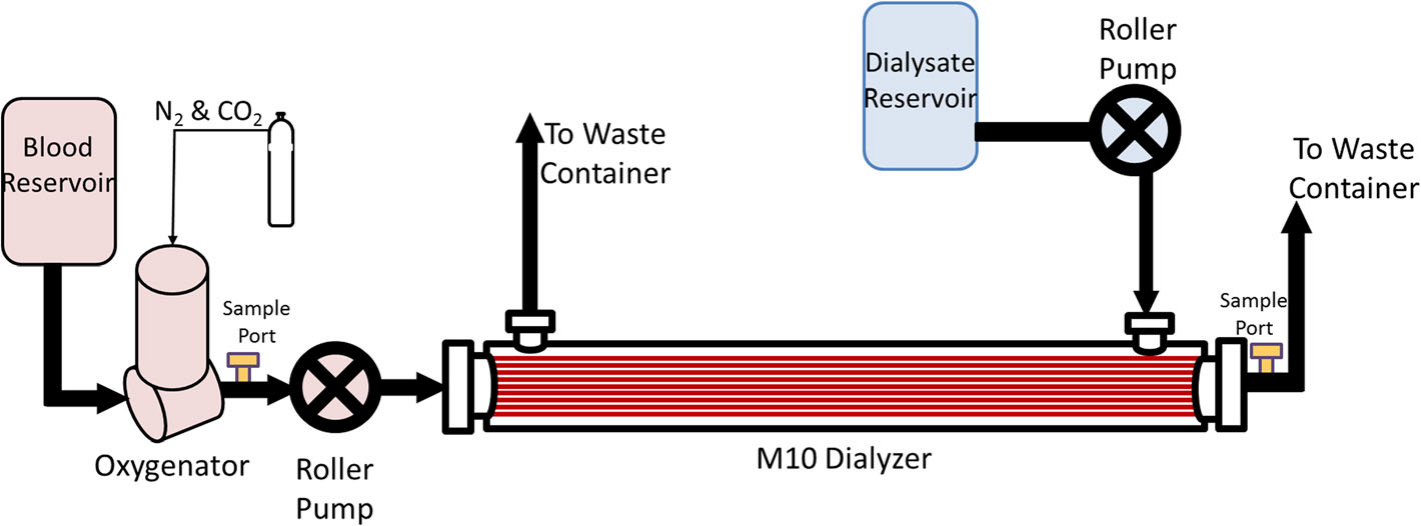
Schematic of the in vitro, single-pass CO_2_ removal setup. Blood was pumped through the inside of the dialyzer fibers while dialysate was independently pumped over the outside of the fibers. The oxygenator conditioned the blood, and blood samples were taken before and after the dialyzer

Gas exchange was tested in a single-pass system (Fig. 1). The blood side of the system consisted of a 6-L reservoir bag, a Medtronic Affinity oxygenator (Medtronic, Minneapolis, MN) used to control inlet pCO_2_ and heat the blood to 37 °C, and a Gambro M10 dialyzer (Gambro, Lyon, Paris; surface area 0.04 m^2^). The dialysis side of the system consisted of a 6-L reservoir bag submerged in a 37 °C water bath. The zero bicarbonate dialysate used is previously described. Masterflex L/S roller pumps (Cole Palmer Instrument Co, Vernon Hills, IL) were used to control blood and dialysate flow rates.

The blood used was same day bovine or porcine blood collected from a local slaughterhouse (Thoma Meat Market, Saxonburg, PA). The blood was heparinized at a concentration of 10 IU/mL and filtered using a 40-μm pore size filter (Pall Biomedical, Inc., Fajardo, PR). Gentamicin was added at a concentration of 0.1 mg/mL to prevent infection. The blood was diluted using 1× phosphate-buffered solution so that the final hemoglobin concentration was 12 ± 1 g/dL and 5% dextrose in 0.45% NaCl (Baxter Healthcare Corp., Deerfield, IL) was added for a final glucose concentration of 100–300 mg/dL.

The benchtop parameters were scaled down based on the surface area ratio of the M100:M10 dialyzers (0.9/0.04), hence a scale factor of 22.5. The Gambro M100 dialyzer would be the dialyzer used in the scaled up system. Blood flow rates were set to 18.7 mL/min (421 mL/min scaled up) and 11 mL/min (248 mL/min scaled up), and dialysis flow rates were varied between 2.2 and 20 mL/min (49.5–450 mL/min scaled up). Blood gases and pH were measured at the inlet and outlet of the dialyzer and analyzed using a Rapid Point 405 Blood Gas Analyzer with Co-oximetry (Siemens Healthcare Diagnostics Inc., Tarrytown, NY). The targeted inlet pCO_2_ was 50 ± 5 mmHg.

Total CO_2_ removal rate was calculated from the change in pCO_2_ and bicarbonate concentration from the inlet to the outlet of the dialyzer, according to Eq. 2.

2 $$ {\overset{.}{V}}_{{\mathrm{CO}}_2}=\left(\Delta \left[{\mathrm{HCO}}_3^{-}\right]+{\Delta \mathrm{pCO}}_2*{K}_{\mathrm{s}}\right)*{Q}_{\mathrm{b}}*{V}_{\mathrm{m}} $$

∆pCO_2_ = change in pCO_2_ across the dialyzer (mmHg), ∆HCO_3_ = change in actual HCO_3_ concentration across the dialyzer (mmol/L), *K*_s_ = CO_2_ solubility constant in blood (0.0307 mmol/mmHg*L) [24], *Q*_b_ = experimental blood flow rate (L/min), and *V*_m_ = molar volume at STP (22.4 mL/mmol). Bicarbonate concentration was calculated using the Henderson-Hasselbalch equation.

## Results

**Fig. 2:**
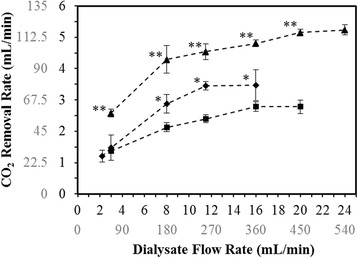
In vitro CO_2_ removal rates. The dual axes show experimental results, as well as the expected values when the system is scaled up by a factor of 22.5. The expected scaled up values are in *gray*, while the actual values are in *black. Triangle symbols*: *Q*_b_ = 11 mL/min (248 mL/min scaled up), pCO_2, inlet_ = 100 mmHg; *diamond symbols*: *Q*_b_ = 18.7 mL/min (421 mL/min scaled up), pCO_2, inlet_ = 50 mmHg; *square symbols*: *Q*_b_ = 11 mL/min (248 mL/min scaled up), pCO_2, inlet_ = 50 mmHg. *Single asterisk* indicates *p* < 0.05 between different blood flow rates (*diamond* and *square symbols*). *Double asterisks* indicate *p* < 0.05 between different inlet pCO_2_ values (*triangle* and *square symbols*)

CO_2_ removal rates measured from respiratory dialysis are shown in Fig. 2 are presented, by a dual axis, in terms of actual and scaled up (actual multiplied by the 22.5 scale factor) CO_2_ removal rates. At an inlet pCO_2_ of 50 mmHg and a blood flow rate of 18.7 mL/min (421 mL/min scaled up), the maximum CO_2_ removal rate was 3.5 ml/min (78 mL/min scaled up), a 24% increase compared to a blood flow rate of 11 mL/min (248 mL/min scaled up) at the same conditions (*p* = 0.048). The effect of doubling the inlet pCO_2_, 50 mmHg to 100 mmHg, was also evaluated at a blood flow rate of 11 mL/min (248 mL/min scaled up) (Fig. 2). There was an 85% increase in CO_2_ removal between the two inlet pCO_2_ conditions.

The blood pH change across the dialyzer is shown in Table 2. No decreases in pH were noted and the largest increase in blood pH was 0.08. This occurred when the inlet pCO_2_ was 100 mmHg. At 50-mmHg inlet pCO_2_, the greatest increase in pH was 0.07 and occurred at a blood flow rate of 11 mL/min and a dialysate flow rate of 16 mL/min. At no point did pH fall as a result of bicarbonate removal with the respiratory dialysis procedure.

**Table 2:** pH of pre- and post-dialyzer blood

Dialysate flow rate (mL/min)		Blood flow rate (mL/min)		
		11 (pCO_2_ = 50 mmHg)	11 (pCO_2_ = 100 mmHg)	18.7 (pCO_2_ = 50 mmHg)
2.2	Pre-dialyzer	- - -	- - -	7.146 ± 0.015^†^
	Post-dialyzer	- - -	- - -	7.151 ± 0.004^†^
3	Pre-dialyzer	7.207 ± 0.030^*^	7.077 ± 0.003	7.144 ± 0.013^*^
	Post-dialyzer	7.230 ± 0.030^*^	7.102 ± 0.010	7.157 ± 0.008^*^
8	Pre-dialyzer	7.189 ± 0.024^*^	7.084 ± 0.008^*^	7.145 ± 0.003^*^
	Post-dialyzer	7.233 ± 0.018^*^	7.130 ± 0.004^*^	7.174 ± 0.002^*^
11.5	Pre-dialyzer	7.197 ± 0.028	7.072 ± 0.013^*^	7.143 ± 0.003^*^
	Post-dialyzer	7.240 ± 0.023	7.125 ± 0.011^*^	7.179 ± 0.005^*^
16	Pre-dialyzer	7.181 ± 0.022	7.086 ± 0.012^*^	7.148 ± 0.019^*^
	Post-dialyzer	7.252 ± 0.023	7.148 ± 0.006^*^	7.181 ± 0.007^*^
20	Pre-dialyzer	7.191 ± 0.024^*^	7.083 ± 0.012^*^	- - -
	Post-dialyzer	7.259 ± 0.027^*^	7.148 ± 0.004^*^	- - -
24	Pre-dialyzer	- - -	7.070 ± 0.008^*^	- - -
	Post-dialyzer	- - -	7.151 ± 0.012^*^	- - -

*p < 0.05, ^†^ *n* < 3

## Discussion

Respiratory hemodialysis, in contrast to traditional ECCO_2_R using a hollow fiber membrane, is an attractive treatment option for patients with hypercarbic respiratory failure as intensive care units already have dialysis equipment available, clinical staff are familiar with its use, lower flow rates, and much reduced air embolism risk. This study demonstrates the use of a novel zero bicarbonate dialysate in a bench scale respiratory hemodialysis system. When the data is scaled up to an adult hemodialyzer, 62–78 mL CO_2_/min was removed without changing blood pH.

The inlet pCO_2_ was doubled, from 50 to 100 mmHg at a constant blood flow rate, in this experiment to evaluate how the CO_2_ removal rates will scale up when the entire system, including dialyzer surface area and flow rates, is scaled up. In the case of the scaled down system tested here, doubling the amount of available CO_2_, accomplished by doubling the inlet pCO_2_, resulted in nearly a doubling of the CO_2_ removed, on average an 87% increase in CO_2_ removal across the range of dialysate flow rates tested. Thus, when the system is scaled up, the scaled up flow rate will deliver 22.5 times more CO_2_ to 22.5 times more dialyzer surface area available for gas exchange, and therefore, the CO_2_ removal rate should increase by the same factor.

Based on contemporary understanding of acid-base balance, previous work by several groups have attempted to replace the bicarbonate with NaOH, Tris, and organic ions [16, 17], believing this would maintain physiological pH. However, these attempts proved unsuccessful, resulting in hemolysis, elevated pulmonary artery pressure, and metabolic acidosis, among other negative effects. Stewart’s approach to acid-base chemistry, used to design the dialysate in this study, places the SID and *A*_tot_ as the most important determinants of pH, whereas bicarbonate concentration is dependent on CO_2_, SID, and *A*_tot_, and therefore does not need to be replaced as along as the strong ion concentration and *A*_tot_ is preserved. The change in blood pH from the inlet to the outlet of the dialyzer is statistically significant for nearly all of the conditions. However, an in vivo respiratory hemodialysis study done by Zanella et al. showed an increase in blood pH of 0.04 between the inlet and outlet of the dialyzer with no reported negative effects [25].

It has been recently demonstrated that CO_2_ removal rates of 40–80 mL/min maintain normocapnia in ARDS patients treated with protective ventilation and partial ECCO_2_R at blood flow rates of 400–500 mL/min [5, 26]. The CO_2_ removal rates achieved in the work described here (63–78 mL/min scaled up) are within clinically useful ranges, and the device was operated at lower blood flow rates (248–421 mL/min scaled up), indicating a higher effectiveness in blood plasma, and a better safety profile. This improved efficiency may reflect the fact that most CO_2_ is transported as bicarbonate ions (>90%), and the greater effectiveness of bicarbonate dialysate is due to bicarbonate rather than dissolved CO_2_ being targeted for removal. During ECCO_2_R using hollow fiber membranes, the CO_2_ held as bicarbonate must be converted to dissolved CO_2_ before it can be removed. This conversion limiting CO_2_ removal rates was demonstrated in a study using hollow fibers immobilized with carbonic anhydrase, to catalyze the conversion of bicarbonate to dissolved CO_2_, resulting in a 37% enhancement of the CO_2_ removal rate [27, 28]. Respiratory hemodialysis, by directly removing bicarbonate, is not limited by the conversion of bicarbonate to dissolved CO_2_ permitting lower blood flow rates yet still able to attain therapeutic results.

Several groups have investigated ways to capitalize on clinician familiarity with dialysis equipment for ECCO_2_R. The PrismaLung uses the PrismaFlex unit as the blood pump for the Medos Hilite oxygenator and demonstrates the efficacy of using dialysis pumps for ECCO_2_R. The PrismaLung system removed 40–60 mL/min of CO_2_ under hypercapnic conditions, at blood flow rates 200–400 mL/min; however, it relies solely on, and is limited by, dissolved carbon dioxide for removal [29]. The respiratory hemodialysis system described in this paper can also use dialysis pumps, but removes 30–58% more CO_2_ at comparable blood flow rates. Zanella et al. have published several respiratory hemodialysis circuits with dialysate recirculation [25, 30]. These systems have used either a membrane lung with a lactic acid infusion or an electrodialysis unit as secondary CO_2_ removal devices in the dialysate. The reported CO_2_ removal rates from the dialysate were 86 and 91 mL/min at a blood flow rate of 250 mL/min, respectively [25, 30].

We recognize that intermittent hemodialysis conditions are not designed for treatment longer than a few hours and a single-pass dialysate may lead to loss of essential minerals, micro-nutrients, hormones, and drugs from the plasma. To prevent this, a less permeable dialyzer could be used or the dialysate could be recycled in a closed loop. The closed loop approach would use a secondary CO_2_ removal device, such as a bubble oxygenator or membrane lung, to remove CO_2_ from the dialysate post-dialyzer and drive the bicarbonate concentration back down towards zero. This approach would be attractive because it would recycle the dialysate and reduce cost. Work on dialysate recirculation by Zanella, previously described, demonstrates therapeutic CO_2_ removal rates and demonstrates that dialysate recirculation for respiratory hemodialysis is possible.

Another limitation of the current bench experiments was the necessity of adjusting dialysate pH to obtain usable measurements with our laboratory blood gas analyzer. For this purpose, hydrochloric acid was added. As a result, the final chloride concentration exceeded 116 mmol/L (Table 1). Consequently, although pH increased slightly in the post-dialyser plasma (Table 2), it did not return to physiologically normal levels of 7.35–7.45, since the chloride resulted in a metabolic acidosis. It should be highlighted, however, that this small pH increase, despite removal of bicarbonate, proving the concept that bicarbonate can be removed without further lowering pH. In future work, we plan to define the safe pH boundaries of our dialysate and target the final dialysate pH accordingly, while limiting chloride, and accomplishing CO_2_ removal with restoration of a physiological pH. Importantly, bicarbonate removal resulted in lower CO_2_ levels and was achieved at much lower blood flows than those required for conventional ECCO_2_R. At these low blood flow rates, about 10% of cardiac output, blood exiting the dialyzer would be significantly diluted by venous blood in circulation.

## Conclusions

ECCO_2_R by continuous hemodialysis is feasible in a bench model of hypercarbic respiratory acidosis without worsening blood pH. There is a critical need for a simple, minimally invasive ECCO_2_R system. Our respiratory dialysis approach promises to fulfill this need, which may have application in a wider cohort of patients including hypercarbic respiratory failure due to asthma, COPD, and restrictive lung disease as well as enable ultra-protective lung ventilation in ARDS.

## Abbreviations

∆HCO_3_: Change in HCO_3_ concentration across the dialyzer
∆pCO_2_: Change in pCO_2_ across the dialyzer
ARDS: Acute respiratory distress syndrome
*A*_tot_: Total concentration of weak acids
CO_2_: Carbon dioxide
COPD: Chronic obstructive pulmonary disease
ECCO_2_R: Extracorporeal CO_2_ removal
HCO_3_^-^: Bicarbonate ion
IHD: Intermittent hemodialysis
*K*_3_: Second dissociation constant of carbonic acid
*K*_a_: Weak acid dissociation constant
*K*_c_: Combined equilibrium and solubility constant for CO_2_
*K*_w_: Autoionization constant of water applied to plasma
LPV: Lung protective ventilation
pCO_2_: Partial pressure of CO_2_
*Q*_b_: Experimental blood flow rate
*S*_a_: Surface area adult dialyzer
*S*_f_: Surface area experimental dialyzer
SID: Strong ion difference
Tris: Tris(hydroxymethyl)aminomethane
VILI: Ventilator-induced lung injury
*V*_m_: Molar volume
